# Osteoarthritic changes rather than age predict outcome following arthroscopic treatment of femoroacetabular impingement in middle-aged patients

**DOI:** 10.1186/s12891-016-1108-6

**Published:** 2016-06-08

**Authors:** Simon Jakob Herrmann, Manuel Bernauer, Benjamin Erdle, Norbert Paul Südkamp, Peter Helwig, Oliver Hauschild

**Affiliations:** Department for Orthopedic and Trauma Surgery, Freiburg University Medical Center, Hugstetterstr. 55, 79106 Freiburg, Germany

**Keywords:** Femoroacetabular impingement, Hip arthroscopy, Alpha angle, Hip outcome score

## Abstract

**Background:**

Our purpose was to evaluate outcome following arthroscopic treatment of femoroacetabular impingement (FAI) in middle-aged patients and to define risk factors for conversion to total hip arthroplasty (THA).

**Methods:**

This was a retrospective case series of 79 consecutive patients (40 to 65 years) undergoing arthroscopic treatment of FAI (follow-up ≥12 months). Outcome at follow-up was assessed using Hip outcome score (HOS). Alpha angle, Kellgren Lawrence grade (K-L grade), joint space width (JS), lateral center edge (LCE) angle, caput-collum-diaphysis (CCD) angle and acetabular index (AI) were analysed retrospectively. THA group and Non-THA group were compared.

**Results:**

Seventy-nine patients (mean age 48.6 years, mean follow-up 32 months) were included. 18 patients (22.8 %) were converted to THA. Mean HOS score in the Non-THA group at time point of follow-up was 80.2. Non-THA group and THA group showed no significant differences for mean age (48.2 years vs. 49.9 years, *p* = 0.278), alpha angel (*p* = 0.541), LCE (*p* = 0.294), CCD (*p* = 0.101) and AI (*p* = 0.661) in contrast to differences for JS (*p* = <0.001) and K-L grade (*p* = <0.001). Risk of conversion to THA was higher for patients with K-L grade 3 (*p* = 0.003) or joint space less or equal 2 mm (*p* = 0.001).

**Conclusions:**

One fifth of the middle-aged patients required early conversion to THA. Advanced JS narrowing and K-L grade rather than age alone can be considered as risk factor for conversion to THA.

**Electronic supplementary material:**

The online version of this article (doi:10.1186/s12891-016-1108-6) contains supplementary material, which is available to authorized users.

## Background

The concept of FAI has become of growing clinical importance and as a result the number of arthroscopic interventions to the hip joint is rising [1]. FAI syndrome was described as a subtle abnormal bone morphology at the femoral head neck junction or the acetabulum causing an intracapsular bony conflict between proximal femur and acetabular rim under motion within the physiological range [2, 3]. This conflict leads to labral tears and abrasion or avulsion of the the cartilage at the acetabular rim and there is evidence that FAI may lead to osteoarthritis (OA) in the long term [2].

Within the last decade, arthroscopic intervention has proven to be an effective treatment for FAI [4, 5]. Arthroscopy allows the surgeon to address the pathological morphology at the femoral head neck junction and the acetabular rim and thereby restore the functional integrity of the hip joint and possibly prevent further degenerative damage to the joint. Prospective studies have shown improving range of motion, pain relief, high satisfaction and gain of function in outcome scores after arthroscopic treatment in the younger population [4, 6, 7].

However, little is known about the outcome in middle-aged patients. It can be assumed that in these patients the conflict between proximal femur and acetabular rim has existed for a longer time and advanced degenerative changes of the cartilage and labrum may already be present at time of intervention [8, 9]. As a consequence this might jeopardize the effects of any intervention aiming at restoration of regular joint anatomy. Some of these patients may therefore not benefit from hip preserving surgery but rather require early conversion to total hip arthroplasty (THA). In an effort to facilitate preoperative decision making it is important to define parameters predicting poor outcome and risk of early conversion to THA [10].

To describe the abnormal head neck offset, the concept of alpha angle was introduced and widely used to define cam impingement [11]. The relation between alpha angle and increased chondral damage and labral injury as well as early progression to THA was described [12, 13]. However, until today there is no study showing correlation between preoperative alpha angle and outcome after arthroscopic treatment.

The aim of this study was to evaluate the mid term outcome following arthroscopic treatment of FAI in middle-aged patients and to define preoperative prognostic factors for early conversion to THA.

## Methods

This was retrospective case series of consecutive patients undergoing arthroscopic treatment of FAI at our institution between January 2009 and August 2012. The study was approved by the local ethical committee (EK 225/13). Minimum follow up time was 12 months. Of the 209 patients operated within this period, 99 met the inclusion criteria, for inclusion and exclusion criteria see Table 1. The indication for surgery was groin pain in combination with radiographic diagnosis of FAI or labral tear detected in magnetic resonance imaging (MRI).

**Table 1:** Inclusion and exclusion criteria

Inclusion criteria	Exclusion criteria
Patients undergoing hip arthroscopy for FAI between 1/2009 and 8/2012 Indication for arthroscopic surgery: groin pain, at least on positive clinical test (FABER, FADIR, anterior impingement test, apprehension test), radiographic signs of FAI (alpha angle > 55° or center of femoral head medial to posterior wall or posterior acetabular wall crosses anterior acetabular wall), labral tear in MRI	Other indication for hip arthroscopy (trauma, ECF, empyema)
Age at time point of surgery between 40 and 65 yrs	
Preoperative radiological diagnostic (plain radiograph in anterior posterior or cross table projection)	No preoperative plain radiograph.
At least 12 mos post hip arthroscopy	

*FABER* Flexion, Abduction, External Rotation, *FADIR* Flexion, Adduction, Internal Rotation, *FAI* femoroacetabular impingement, *yrs* years, *mos* months, *ECF* Epiphyseolysis capitis femoris

Three surgeons highly experienced in arthroscopic intervention were involved in the surgical treatment. Arthroscopic intervention was performed with the patient in supine position on a fracture table under general anesthesia and antibiotic prophylaxis with cefuroxime.

Cam impingement was treated by femoral osteochondroplasty. Synovial debridement was performed in cases with synovitis. Labral tear was treated by partial resection of the labrum or labral repair with suture anchors at the surgeon’s discretion. Acetabular overcoverage was treated with acetabular rim trimming and refixation of the labrum. Postoperative partial weight bearing with 20 kg was indicated for 3 weeks after femoral osteochondroplasty and for 6 weeks after labral repair. Flexion of the hip joint was restricted to 90° for 3 weeks postoperatively. All patients were referred to outpatient physiotherapy. Indomethacin was administerd for 14 days postoperatively for all patients to prevent heterotopic ossification.

Upon institutional review board approval conventional radiographs were analyzed retrospectively by one observer (SJH). Alpha angle was assessed in cross-table lateral view [11]. Lateral-center-edge (LCE) angle, caput-collum-diaphysis (CCD) angle and acetabular index (AI) were measured in cross-table lateral radiographs [14]. Joint space width (JS) was measured as the narrowest distance between acetabulum and femoral head at three points (superior lateral edge, middle of the sourcil, at the border to the fovea). All measurements were performed using the digital caliper system of OfficePACS system with an accuracy of one decimal place. All radiographs were graded using the Kellgren Lawrence classification [15].

At time point of follow up patients were examined by telephone interview. Further surgery done to the hip in question including conversion to THA and time point of reoperation was recorded. We asked for the subjective change in joint function following intervention on a scale from−100 % (representing maximum of deterioration) to +100 % (representing maximum of improvement). Level of satisfaction with the surgical intervention and outcome was categorized from 1 (completely unsatisfied) to 4 (completely satisfied). Patient were asked, whether they would retrospectively opt for hip arthroscopy as a treatment option again. Additionally, the German version of the hip outcome score (HOS) and the hip outcome score sports subscale (HOSS) were assessed for all patients without conversion to THA.

### Statistical analyses

Survivorship was defined as preservation of the native hip joint until follow-up. Patients who had undergone conversion to THA in the time between index operation and follow-up (THA group) were compared to patients who did not require conversion to THA (Non-THA group). Kolmogorov-Smirnov test confirmed normally distributed outcome data for both groups. A parametric *T*-test for independent samples was used to compare age and radiographic parameters between THA and Non-THA groups.

To determine the effect of osteoarthritic changes on outcome patients were divided in groups with Kellgren Lawrence (K-L) grades 0, 1 or 2 and K-L grade 3 and JS > 2 mm and JS ≤ 2 mm, respectively and frequency tables were analysed using Fisher’s and Chi-square tests. Spearman’s rho correlation coefficient was used to describe correlations between categorical and continuous, normally distributed data. Pearson correlations were used to detect associations between continuous, normally distributed variables. All reported *p*-values are two tailed with an alpha level of 0.05 indicating significance.

SPSS Version 21 (SPSS Inc, Chicago, IL, USA) was used for all statistical analysis.

## Results

Seventy-nine patients (80 %) out of 99 patients fulfilling inclusion criteria were available for follow up interviews. Mean follow up period was 32 months. Mean age at time of index surgery was 48.6 (Table 2).

**Table 2:** Descriptive statistics

Follow up period (min 12 mos)	32.3 (SD ± 12.9) mos
Age at time of index surgery	48.6 (SD ± 6.1) yrs
Conversion to THA	18 patients (22.8 %)
Survivorship group THA	min 5 mos, max 45 mos

*THA* total hip arthroplasty, *yrs* years, *mos* months, *SD* standard deviation, *min* minimum, *max* maximum

**Fig. 1:**
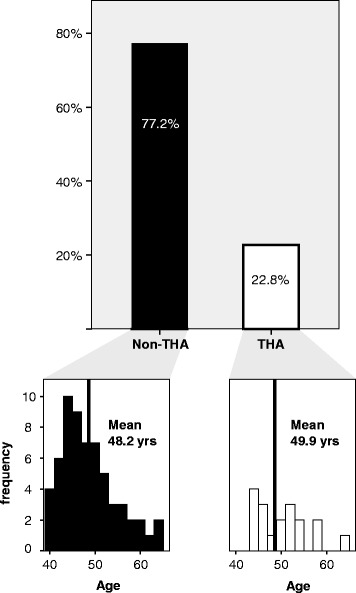
Risk of conversion to THA, age distribution and mean age in THA and non THA-group. THA total hip arthroplasty, yrs years

**Fig. 2:**
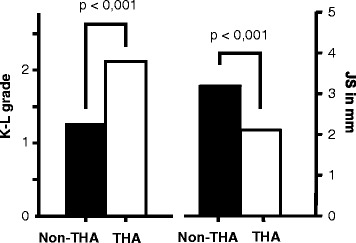
Mean Kellgren Lawrence grade and joint space width for non-THA and THA group. *T*-test reveals significant differences. THA total hip arthroplasty, K-L grade Kellgren Lawrence grade, JS Joint space width

Eighteen patients (22.8 %) underwent conversion to THA within the follow up period. Minimum survivorship in the Non-THA group was 5 month and maximum was 45 months. Non-THA group and THA group were comparable with regard to age and all preoperatively assessed angles (Table 3, Fig. 1). However, patients with K-L grade of 3 were significantly more likely to require THA following arthroscopic intervention as compared to those with K-L grade 2 or less (66.7 vs. 16.2 %, *P* = 0.003). The same was true for patients with a JS of 2 mm or less as compared with JS > 2 mm (75 vs. 15.9 %, *P* = 0.001). Data are summarized in Tables 3 and 4 and Fig. 2.

**Table 3:** Mean values of patient characteristics and T-test comparison of non-THA and THA group

		All	Group non-THA	Group THA	*P* value^a^
Number of patients		79	61 (77.2 %)	18 (22.8 %)	
Male/female		49/30	38/23	11/7	
Age at surgery in years	(SD)	48.6 (±3.3)	48.2 (±6.2)	49.9 (±5.6)	0.278
K-L grade		1.45 (±0.8)	1.27 (±0.8)	2.12 (±0.8)	<0.001^*^
JS in mm		3.3 (±1.0)	3.5 (±0.8)	2.3 (1.1)	<0.001^*^
Alpha angle (°)		67 (±13)	67.5 (±12.6)	64.7 (±15)	0.541
LCE angle (°)		32 (±7.5)	32 (±7.3)	31.5 (±8.7)	0.77
CCD angle (°)		130.9 (±5.4)	131 (±5.6)	130.8 (±5.1)	0.92
AI (°)		6.6 (±5.6)	6.3 (±5.6)	7.4 (±5.5)	0.511

*THA* total hip arthroplasty, *K-L* grade Kellgren Lawrence grade, *JS* joint space width, *LCE* lateral center edge angle, *CCD* caput-collum-diaphysis angle, *AI* acetabular index, *SD* standard deviation

^*^Significant at 0.05 level

^a^ *T*-test

**Table 4:** Increasing percentage of patients requiring conversion to THA in dependence of preoperative advanced joint space narrowing and high K-L grade

		Non-THA	THA
		*N*	*N* (%)
JS in mm	>4	20	1 (4.8)
	>2, ≤ 4	38	10 (20.8)
	≤2	2	6 (75)
	>2	58	11 (15.9)
K-L grade	0	8	0 (0)
	1	31	4 (11.4)
	2	18	7 (28)
	3	3	6 (66.7)
	Group 0, 1, 2	57	11 (16.2)

*THA* total hip arthroplasty, *JS* Joint space width, *K-L* Kellgren Lawrence

Accordingly, 22.8 % of patients from THA group would retrospectively choose for arthroscopic treatment again, as compared to 78.8 % from the Non-THA group (*p* < 0.001, Chi Square). However, within the THA group time of survivorship did not significantly correlate with differences between patients who would chose arthroscopic treatment again and the patients who would refuse (rho = 0.335, *P* = 0.174). In the THA group we did not see a correlation between the decision in favour of arthroscopic treatment and survivorship (rho = 0.191, *P* = 0.447).

Mean HOS score in the Non-THA group at time point of follow up was 80.2 (±16.2) and mean HOSS was 63.2 (±24.8). Mean subjective change in function in the Non-THA group was + 54.3 % (±41.5 %), mean patient related satisfaction 3.2 (±0.9) (Table 5). HOS did not correlate with any of the preoperatively assessed angles (Pearson’s correlation coefficient−0.04 < *r* < 0.21).

**Table 5:** Outcome scores and subjective outcome parameters

Characteristic	Mean (SD)
HOS	80.2 (±16.2)
HOS Sport	63.2 (±24.8)
Subjective change in function (−100 % to +100 %)	+54.3 % (±41.5)
Patient related satisfaction 1 (completely unsatisfied) to 4 (completely satisfied)	3.2 (±0.9)

*HOS* Hip outcome score, *SD* standard deviation

We saw two revision hip surgeries because of treatment failure (one open revision surgery and microfracturing, one arthroscopic revision surgery and labral repair) and one surgery because of major complication (1.3 %, osteosynthesis of insidious femoral neck fracture).

## Discussion

The aim of this study was (1) to evaluate the mid-term outcome and patient satisfaction following arthroscopic treatment of FAI in middle-aged patients and (2) to define preoperative radiographic factors predictive for outcome. The most important findings of this study were that (1) age alone should not be seen as contraindication for successful treatment of FAI and reasonable outcome can be expected for the majority of patients and (2) a preoperative K-L grade 3 and/or a joint space width of less than 2 mm will be associated with high rates of early conversion to THA and poor patient satisfaction with arthroscopic intervention.

Appreciation of the limitations of this study is warranted. It is a strength of this study that it is based on a consecutive series of patients. However, this is an analysis of retrospectively collected data with inherent limitations typical of retrospective studies. First and foremost, there was no control group and preoperative scores were not available. Yet, THA and non-THA groups were comparable with regard to demographic parameters. Owing to the lack of preoperative scores we cannot exclude a difference in preoperative functional impairment inbetween groups. In this regard our study can not give an answer to the question whether there is a threshold in preoperative functional scoring associated with an elevated risk for early conversion to THA.

The validity of the HOS may have been compromised by the assessment in telephone interview. Nevertheless, the outcome measurements were comparable to those reported in the literature.

There was a limited number of patients included in the study, ending up with only 18 patients in the THA group. Comparable to other studies reporting on arthroscopic treatment of FAI, the follow up time was limited and the follow up period ranged from 12 to 55 months. Still, the mean follow up of the present study was almost 3 years with a maximum of 45 months. Thus, we can only report on midterm outcome, the number of conversions to THA in the long run cannot be derived from our data and remains unclear. Owing to the retrospective nature of the study follow-up varied widely from 12 to 45 months. This should be kept in mind when interpreting the results. Moreover, follow-up was significantly shorter in the non-THA as compared to the THA group (30.9 vs. 39.0 months, *P* = 0.02). This might bias the results in favour of the non-THA group. It is not unreasonable to assume, that the rate of conversions to THA would have increased with equal follow-up. Given the strong association of Kellgren-Lawrence grade and joint space narrowing found in the present analyses we, however, hypothesize that patients with advanced degenerative changes would be most likely to fall from the non-THA into the THA group rather enhancing than jeopardizing the robustness of our results. Yet, in the end this remains speculative.

Finally, 20 patients eligible for inclusion in the study were unavailable for follow-up. Even though only three patients specifically declined participation in the study, a potential bias introduced by loss to follow-up cannot fully be excluded. A follow-up rate of 80 % as achieved in the present study is, nevertheless, generally considered acceptable for retrospective analyses [16].

Arthroscopic treatment of FAI has proven to show significant improvement in outcome scores and effective pain relief in a young, active population [6, 17]. However, little is known about outcome in the older population [18]. In our study, most of the patients showed at least slight radiological signs of OA of the hip (Table 4). Not surprisingly, 22.8 % of the patients included into the study required conversion to THA during follow up period, which is more than it was described for younger populations [4]. This reflects the advanced damage to the hip joint in an older population with FAI leading to failure after arthroscopic treatment [9]. We found that in our study population joint space equal or less 2 mm and K-L grade more than 2 were strong predictive factors for conversion to THA. This is consistent with a study by Philippon et al., first defining joint space narrowing 2 mm or less and K-L grade 3 or 4 as a predictor of conversion to THA in 80 and 73 % of the cases [10].

Surprisingly we did not see a correlation between age and risk of conversion to THA for patients between 40 and 65 years of age. The mean age in the THA and the Non-THA groups were comparable. In contrast to our study group of middle-aged patients risk for early conversion to THA reported in the literature for adolescent or young patients without signs of advanced OA effectively approaches zero [4, 7]. However, in our analysis age was not a predictive factor for conversion to THA. Taking into account our results, advanced age itself should not be considered as a contraindication for arthroscopic treatment in patients with FAI.

Early conversion to THA represents, that some patients did not benefit from arthroscopic treatment. However, survivorship within these patients ranged between 5 and 45 months. One could argue, that arthroscopic treatment may have been effective in delaying time to THA at least for some patients. After all, more than one quarter of patients with conversion to THA expressed that they would opt for arthroscopic intervention again. The individual decision did, however, not correlate with time of survivorship. There is still considerable debate on effectiveness of arthroscopic treatment of OA of the hip. Some authors report good outcome after arthroscopic debridement and lavage whereas others see poor results [19–22]. However, one should keep in mind, that outcomes after arthroscopic lavage or arthroscopic debridement for OA of the knee were no better than those after a placebo procedure [23].

The HOS was introduced and validated to measure outcome after treatment of FAI [24]. Until today the numbers of publications measuring functional outcome using HOS is limited [5, 6, 25]. We reported satisfactory outcome evaluated in HOS and HOSS in the Non-THA group. HOS showed strong correlations with subjective satisfaction (data not shown). In a recent study of 52 patients with a slightly younger study population (median age 42 years) comparable outcome was reported [25]. However, several studies reported superior outcome scores compared to ours in in the young and active population [6, 17]. Taking into consideration the predictive factors joint space and K-L grade as discussed above, early conversion to THA can be avoided and good outcome can be expected even in mature patients.

Besides clinical tests impingement syndrome is usually diagnosed by defining the pathological cam or pincer morphology in conventional radiographs using alpha angle, LCE or AI [11, 26]. The alpha angle was introduced to describe the abnormal head neck offset and is used to define cam impingement when exceeding 55°. Several recent studies indicate that this border is rather arbitrary [27–29]. In a recent CT-study 36 out of 100 asymptomatic subjects were found to present with alpha angle more than 55° [27–29]. Mean alpha angle in our symptomatic study population was 67°. Neither could we detect a significant difference in alpha angle between the THA group and the Non-THA group nor was the alpha angle correlated with outcome measured by HOS in the Non-THA group. Alpha angle has been shown to be correlated with age, labral tears and chondral defects [28, 30, 31]. However, our data demonstrate that the outcome after hip athroscopy could not be predicted by preoperative alpha angle. The pathological hip morphology in FAI is too subtle and complex to be described by one single parameter in plain radiographs [29, 31]. Since clinical tests for FAI have shown to be of limited diagnostic value the diagnosis of FAI based on physical examination and plain radiographs remains challenging [32–34]. In an effort to improve preoperative diagnostic accuracy of FAI and possible additional pathologies future analyses aiming at diagnostic value of contrast-enhanced MRI and quantitative assessment of cartilage composition are warranted. Further studies to identify risk factors for treatment failure and early conversion to THA in mature patients are needed.

## Conclusion

Our data show that advanced joint space narrowing and K-L grade should be considered as contraindication for arthroscopic treatment. Taking into account these contraindiacations good mid-term outcome can be expected in middle-aged patients.

## Abbreviations

AI, acetabular index; CCD, caput-collum-diaphysis; FABER, flexion, abduction, external rotation; FADIR, flexion, adduction, internal rotation; FAI, femoroacetabular impingement; HOS, hip outcome score; HOSS, hip outcome score sports; JS, joint space width; K-L grade, kellgren lawrence grade; LCE, lateral center edge; max, maximum min: minimum; mos, Months; MRI, magnetic resonance imaging; OA, osteoarthritis; SD, standard deviation; THA: THA; Yrs: years

## Additional files

Additional file 1: Telephone interview, template for telephone interview in german. (PDF 65 kb) Additional file 2: Dataset, dataset in tabular form. (PDF 39 kb)
