# Supplementary material for: Osteoarthritic changes rather than age predict outcome following arthroscopic treatment of femoroacetabular impingement in middle-aged patients
Source: BMC Musculoskelet Disord. 2016 Jun 8;17:253. doi: 10.1186/s12891-016-1108-6 (PMC4898370; doi:10.1186/s12891-016-1108-6)
Supplement: Additional file 1: — Telephone interview, template for telephone interview in german. (PDF 65 kb) [file 12891_2016_1108_MOESM1_ESM.pdf]

Telefoninterview

Telefonische Aufklärung über die Studie:

Ziel der Studie: Auswertung der bisher durchgeführten Hüftarthroskopien bei Hüftimpingement um das postoperative Ergebnis verschiedener Altersgruppen zu vergleichen. Anhand der vorliegenden Unterlagen sollen Faktoren ermittelt werden, die Einfluss auf das postoperative Ergebnis haben. Ziel ist es damit, für zukünftige Patienten die Erfolgsaussichten mit Hüfarthroskopie genauer abzuschätzen.

Methodik: Kurzes Telefoninterview von ca. 5min Dauer. Auswertung der bereits vorliegenden Unteralgen und Daten: OP-Berichte, Röntgenbilder, Arztbriefe. Die Daten werden für die Auswertung anonymisiert.

Zur Studienteilnahme: Teilnahme ist freiwillig, es entstehen keine Vorteile für Teilnehmer an der Studie. Neben dem Telefoninterview keine weiteren Befragung oder Untersuchung. Alle Daten werden für die Auswertung anonymisiert. Einverständnis kann jederzeit widerrufen werden ohne Einfluss auf die weitere Behandlung in unserer Klinik. Bestehen noch Fragen zur Studie oder zur Teilnahme?

1. Patient erklärt sein Einverständnis zur Teilnahme an der retrospektiven Studie.

   O Ja O Nein

2. Weitere Operationen an der von uns operierten Hüfte? Bei weitere Operation: Wann und was wurde operiert (weitere HASK oder HTEP?)

3. Subjektive Veränderung der Funktion der Hüfte im Vergleich zu vor der Operation von -100% (maximale Verschlechterung) bis +100% (maximale Verbesserung)

„Wie wurden ihre Beschwerden mit der Hüfte durch die Operation auf einer Skala von -100% bis +100% bis heute beeinflusst, wenn -100% die maximale Verschlechterung, 0% ein Gleichbleiben der Beschwerden und 100% die maximale Verbesserung darstellt.“

Bei Z.n. weiterer Operation:

„Wie wurden ihre Beschwerden mit der Hüfte durch die Operation auf einer Skala von -100% bis +100% bis zur der weiteren Operation beeinflusst, wenn -100% die maximale Verschlechterung, 0% ein gleich bleiben der Beschwerden und 100% die maximale Verbesserung darstellt.“

4. <u>Subjektive Zufriedenheit mit Verlauf nach Operation</u>

„Wie zufrieden sind sie insgesamt mit dem Verlauf nach der Operation bis heute: Sehr zufrieden, zufrieden, wenig zufrieden, nicht zufrieden.“

O Sehr zufrieden O Zufrieden O Wenig zufrieden O Nicht zufrieden

„Mit dem Wissen über den Verlauf (Behandlungsergebnis und perioperative Einschränkungen), würden Sie den Eingriff nochmals durchführen lassen?“

<u>O Ja</u> <u>O Nein</u>

5. <u>HOS Score inkl. HOSS</u>

Hüft Outcome Score (HOS)

„Bitte beantworten Sie jede Frage mit der Antwort, die Ihren Zustand während der letzten Woche am besten beschreibt. Falls die betreffende Aktivität durch etwas anderes als durch Ihre Hüfte eingeschränkt ist, antworten Sie bitte mit ‚Nicht zutreffend (NZ)‘*.“*

	Keinerlei Schwierigkeiten	Geringe Schwierigkeiten	Mässige Schwierigkeiten	Extreme Schwierigkeiten	Unmöglich	NZ
1) 15 Minuten stehen						
2) In ein gewöhnliches Auto ein- und aussteigen						
3) Socken und Schuhe anziehen*						
4) Steile Hügel hinauf gehen						
5) Steile Hügel hinunter gehen						
6) Eine Etage hinauf gehen						
7) Eine Etage hinunter gehen						
8) Bordsteine rauf und runter gehen						
9) Tief in die Hocke gehen						
10) In eine oder aus einer Badewanne steigen						
11) 15 Minuten sitzen						
12) Die ersten Schritte Gehen						
13) Etwa 10 Minuten gehen						
14) 15 Minuten oder länger gehen						
15) Sich auf dem betroffenen Bein drehen						
16) Sich im Bett umdrehen						
17) Leichte bis mittelschwere Arbeit (Stehen, Gehen)						
18) Schwere Arbeit (Schieben, Ziehen, Klettern, Tragen)						
19) Freizeitaktivitäten						

Hüft Outcome Sport Score (HOSS)

	Keinerlei Schwierigkeiten	Geringe Schwierigkeiten	Mässige Schwierigkeiten	Extreme Schwierigkeiten	Unmöglich	NZ
S1) 1-2 km Joggen						
S2) Springen/Hüpfen						
S3) Gegenstände wie z. B. einen Golfschläger schwingen						
S4) Landen						
S5) Schnell losrennen und abstoppen						
S6) Hacken schlagen/seitliche Bewegungen						
S7) Wenig belastende Aktivitäten wie schnelles Gehen						
S8) Aktivitäten so ausüben, wie Sie es normalerweise tun						
S9) Einen gewünschten Sport so lange ausüben, wie Sie es wollen						

6. Gab es Komplikationen?

a) Keine
b) Wundheilungsstörung/Infekt
c) Fraktur
d) Nervenschaden temporär (ggf. welche)
e) Nervenschaden dauerhaft (ggf. welche)
f) Sonstige

7. Wenn ja, war eine Revisionsoperation wegen Komplikationen erforderlich?

a) Ja, ggf. welche
b) nein
