# Supplementary material for: Osteoarthritic changes rather than age predict outcome following arthroscopic treatment of femoroacetabular impingement in middle-aged patients
Source: BMC Musculoskelet Disord. 2016 Jun 8;17:253. doi: 10.1186/s12891-016-1108-6 (PMC4898370; doi:10.1186/s12891-016-1108-6)
Supplement: Additional file 2: — Dataset, dataset in tabular form. (PDF 39 kb) [file 12891_2016_1108_MOESM2_ESM.pdf]

No°	THA	Age	Follow up	Survival	Change	Satisfaction	ChooseAgain	HOS	HOSS	alphaAngle	CCD	LCE	AI	JS	KL grade
	0-nonTHA	years	months	months			0-No			preOP					
	1-THA						1-Yes			°	°	°	°	mm	
1	1	44	53	23	-100	1	0				130	31	4	2,3	2
2	1	52	49	45	0	4	0			93	134	22	15	3,2	2
3	1	44	52	38	0	4	0			68	124	21	16	0	3
4	1	44	52	20	0	2	1				133	39	6	2,7	2
5	1	49	51	26	0	4	0			59	124	32	3	4,8	1
6	1	46	48	14	50	2	0			44	137	42	1	3,4	1
7	1	50	47	37	60	3	0			65				2,4	2
8	1	47	47	16	-70	4	0				127	27	11	2,2	1
9	1	43	46	23	20	2	0				130	20	5	1,2	3
10	1	63	36	5	100	4	1			60	130	35	6	1,7	3
11	1	46	39	12	0	3	1				129	23	6	0,8	3
12	1	54	37	6	0	3	0			58	137	45	2	2,6	3
13	1	46	32	19	-90	1	0				140	22	15	2,3	1
14	1	51	31	12	50	3	0				131	34	10	1,8	2
15	1	53	27	8	100	3	1			87	137	40	10	4	2
16	1	57	16	10	-100	1	0			67	131	31	12	2,1	3
17	1	52	20	16	90	3	1			57	123	25	7	1,9	2
18	1	58	19	5	0	1	0			54	126	46	-4	2,7	2
19	0	42	55	55	70	3	1	74	57	51	130	35	4	1,9	2
20	0	43	53	53	50	4	1	79	39	81	134	32	2	2,5	3
21	0	43	48	48	50	2	1	97	44		125	29	11	2,9	2
22	0	50	49	49	100	4	1	100	100		126	19	11	3,7	1
23	0	40	49	49	40	3	1	75	47		130	38	5	3,8	2
24	0	52	48	48	20	2	0	57	22	54	134	13	20	4,2	1
25	0	45	47	47	100	4	1	100	100	76	131	34	7	3,4	2
26	0	47	46	46	100	2	0	72	39	51	125	32	5	3	0
27	0	45	46	46	40	4	1	87	36		127	31	10	3,8	2

28	0	56	41	41	0	2	0	74	39	94	136	32	14	3,7	1
29	0	40	45	45	80	4	1			62	129	25	9	3,4	1
30	0	46	45	45	40	4	1	96	66	61	134	24	5	4,4	1
31	0	46	44	44	0	4	1	68	22	55	126	27	8	4,6	2
32	0	43	43	43	100	4	1	96	100	76	139	41	5	2,1	1
33	0	47	43	43	-20	4	1	87	72		118	28	6	3,4	2
34	0	45	43	43	50	4	1	82	50	90	140	24	6	3,8	1
35	0	41	42	42	-100	3	0	41	31		128	28	7	3,3	1
36	0	64	36	36	40	3	1	87	81	64	134	34	6	3,3	1
37	0	43	41	41	0	1	0	76	83	59	132	32	1	4,7	1
38	0	41	41	41	50	3	1	84	50	67				3,5	1
39	0	50	41	41	40	3	1	72	50	48	125	32	5	2,8	1
40	0	55	41	41	100	4	1	76	60	83	135	14	24	3,6	2
41	0	44	41	41	50	4	1	79	39	68	132	28	6	2,4	3
42	0	62	33	33	30	3	1	57	28	72	140	41		4,2	2
43	0	47	37	37	0	3	1	71	47	54	138	41	3	4,3	0
44	0	58	37	37	0	3	0	41	28		127	37	7	1,9	2
45	0	45	32	32	80	3	1	97	56	70	137	32	7	4,9	2
46	0	46	30	30	100	4	1	97	64	85	143	36	8	4,1	1
47	0	40	34	34	90	4	1	99	100	78	124	31	3	2,7	1
48	0	49	33	33	80	3	1	87	94	75	135	25	10	2,4	3
49	0	44	32	32	80	3	1	94	100	55	129	22	13	4,9	0
50	0	60	25	25	50	2	1	63	31	74	129	35	11	2,8	1
51	0	45	26	26	50	4	1	93	83	77	142	33	8	2,8	2
52	0	41	30	30	70	3	1	94	88	61	140	34	4	4,8	1
53	0	49	28	28	50	3	1	62	28	65	136	39	0	3,4	2
54	0	48	23	23	100	3	1	87	42	70	129	19	12	4,3	0
55	0	43	27	27	60	4	1	65	67	78	129	48	-2	4,1	1
56	0	45	23	23	20	2	0	63	53	76	135	37	2	2,9	2
57	0	52	26	26	60	3	1	85	75		124	45	1	3,6	2
58	0	50	21	21	75	3	1	76	78	46	133	19	18	2,6	1
59	0	42	24	24	100	3	1	91	78		128	36	-2	5	0
60	0	60	18	18	100	3	1	82	89	78	138	32	7	4,7	2

61	0	47	22	22	100	4	1	100	86		140	39	1	3,4	1
62	0	52	22	22	0	1	0	47	44		125	29	10	3,7	1
63	0	63	17	17	100	4	1	100	100	41	126	31	14	4,6	1
64	0	49	22	22	0	4	0	86	58	69	129	31	9	4,5	0
65	0	41	17	17	100	4	1	100	100	78	129	40	3	3,1	1
66	0	58	16	16	70	4	1	95	81	60	133	42	1	2,4	2
67	0	49	21	21	100	4	1	99	94	83	128	26	12	2,6	1
68	0	43	17	17	80	3	1	76	47	70	132	38	4	2,7	1
69	0	44	19	19	-30	2	0	75	56	85	122	33	3	3,3	0
70	0	56	18	18	10	4	0	72	68	49	126	32	11	3,6	1
71	0	52	18	18	70	3	1	48	31	50	124	39	-1	4	1
72	0	53	18	18	50	4	1	52	36	75	119	34	7	2,8	2
73	0	48	15	15	70	3	1	65	53	59	129	33	1	3	1
74	0	44	15	15	70	4	1	94	89	75	129	46	-4	3	1
75	0	40	14	14	100	3	1	97	100	73	130	35	8	4,6	1
76	0	53	13	13	30	2	0				131	24	10	3,4	1
77	0	51	12	12	80	3	1	87	81	69	130	32	-2	4,2	1
78	0	53	12	12	20	1	0			56	131	30	6	4	0
79	0	48	12	12	100	4	1	94	86	60	136	37	-6	4,1	1
